# Nanoimprint-induced strain engineering of two-dimensional materials

**DOI:** 10.1038/s41378-024-00669-6

**Published:** 2024-04-08

**Authors:** Chuying Sun, Jianwen Zhong, Zhuofei Gan, Liyang Chen, Chuwei Liang, Hongtao Feng, Zhao Sun, Zijie Jiang, Wen-Di Li

**Affiliations:** The University of Hong Kong, Hong Kong, China

**Keywords:** Electronic properties and materials, Structural properties

## Abstract

The high stretchability of two-dimensional (2D) materials has facilitated the possibility of using external strain to manipulate their properties. Hence, strain engineering has emerged as a promising technique for tailoring the performance of 2D materials by controlling the applied elastic strain field. Although various types of strain engineering methods have been proposed, deterministic and controllable generation of the strain in 2D materials remains a challenging task. Here, we report a nanoimprint-induced strain engineering (NISE) strategy for introducing controllable periodic strain profiles on 2D materials. A three-dimensional (3D) tunable strain is generated in a molybdenum disulfide (MoS_2_) sheet by pressing and conforming to the topography of an imprint mold. Different strain profiles generated in MoS_2_ are demonstrated and verified by Raman and photoluminescence (PL) spectroscopy. The strain modulation capability of NISE is investigated by changing the imprint pressure and the patterns of the imprint molds, which enables precise control of the strain magnitudes and distributions in MoS_2_. Furthermore, a finite element model is developed to simulate the NISE process and reveal the straining behavior of MoS_2_. This deterministic and effective strain engineering technique can be easily extended to other materials and is also compatible with common semiconductor fabrication processes; therefore, it provides prospects for advances in broad nanoelectronic and optoelectronic devices.

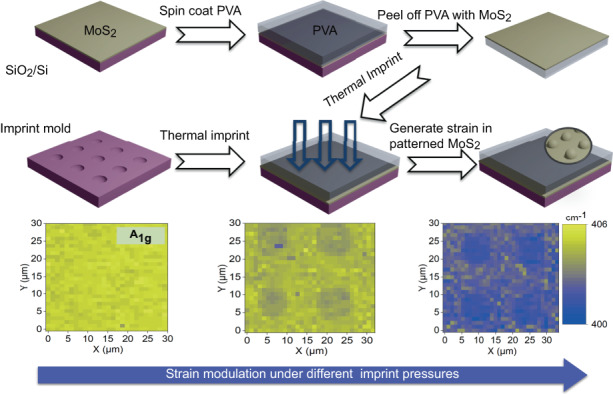

## Introduction

Recently, there has been a strong resurgence of interest in 2D materials owing to their outstanding optical, electrical, and mechanical properties^1,2^. To meet the versatile demands of various applications, several attempts have been made to tailor the properties of 2D materials, by using electrostatic gating^3,4^, chemical doping^5,6^, and hydrogen absorption^7,8^. However, the device’s performance remains unsatisfactory. Exploiting the high flexibility of 2D materials^9,10^, inducing mechanical deformation to alter the crystal structure by strain engineering is considered a promising method to modulate material properties^11–13^. The infusion of strain has been shown to significantly impact key transport properties of 2D materials, such as carrier mobility^14^, conductivity^15^, and band gap modulation^16^. Benefiting from strain-tuned properties, 2D material-based devices have also shown improved performance, as evidenced by enhanced photoresponsivity^17^ and improved carrier mobilities^18,19^. Moreover, emerging studies show that out-of-plane strain, which involves the deformation perpendicular to the plane of 2D materials^20,21^, can precisely alter interlayer exciton properties, revealing the significant potential for the advancement of excitonic devices^22–24^. These recent breakthroughs indicate that strain engineering of 2D materials will stimulate novel and exciting technological advances.

Although various types of methods have been utilized to deform 2D materials, both the magnitude and distribution of the strain cannot be precisely controlled. Deforming flexible substrates transferred with 2D materials is considered a common method^25,26^ but often results in limited strain magnitude due to weak van der Waals (vdW) forces at the interface^27^. Increasing research initiatives are being directed toward the creation of spatially inhomogeneous strain in 2D materials by transferring 2D materials to a non-flat substrat^28,29^. However, this method risks damaging the materials during the transfer process and suffers from uncontrollable strain magnitudes. To gain better control over the magnitudes and distributions of strain in 2D materials, recent studies have focused on the direct patterning of substrates after transferring 2D materials. Various straining techniques, including probe-based indentation via atomic force microscopy (AFM)^30,31^, laser direct writing^32,33^, and electron beam lithography^34^, have also been utilized to produce a controllable strain pattern. However, these methods often suffer from high cost and low throughput and are limited by their use of a small area. Since nanoimprint lithography is a scalable, cost-effective fabrication method capable of fabricating high-resolution nanostructures, it has recently been used to facilitate the low-cost and rapid generation of large-scale strain profiles in 2D materials^35,36^. However, this method also faces obstacles, particularly upward folding or damage to 2D materials and a reduced effective imprint due to the elastic response of the polymer upon mold withdrawal. Therefore, the use of strain to manipulate 2D material properties still depends on an effective strain engineering method that integrates precision, reliability, scalability, and controllability and is suitable for ultimate application.

In this study, we report an effective and deterministic strain engineering strategy for 2D materials performed by nanoimprint lithography, which is termed the nanoimprint-induced strain engineering (NISE) method. We demonstrate this method by manipulating a monolayer of MoS_2_ on polyvinyl alcohol (PVA) to conform to an imprint mold with predetermined dimensions. The strain profile of MoS_2_ is subsequently replicated from the imprint mold, leading to precisely and spatially controllable strain patterns over large areas. In contrast to previous nanoimprint-related methods, our method eliminates the need for a separation process, which could substantially restrict the elastic response of the polymer, and effectively prevents the 2D materials from folding upwards. Consequently, this enables a reduction in damage and preservation of the strain induced in 2D materials. Moreover, our approach eliminates the need for external setups to maintain strain; thus, it is compatible with semiconductor fabrication and ideal for device integration. Our technique also allows for varying strain magnitudes and patterns to be introduced to 2D materials by carefully tailoring the mold’s dimensions and the pressure applied during imprinting. Raman and photoluminescence (PL) spectroscopy were used to evaluate the different strain profiles on MoS_2_. The straining behavior of MoS_2_ during the NISE process was further studied by performing 3D finite element analysis. Furthermore, our method could be extended to other 2D materials with various strain profiles. It is expected that this new strain engineering method will facilitate a new path for strain engineering and improve the applications of 2D materials.

## Results and discussion

### Elastic straining of MoS_2_ by the NISE method

**Fig. 1:**
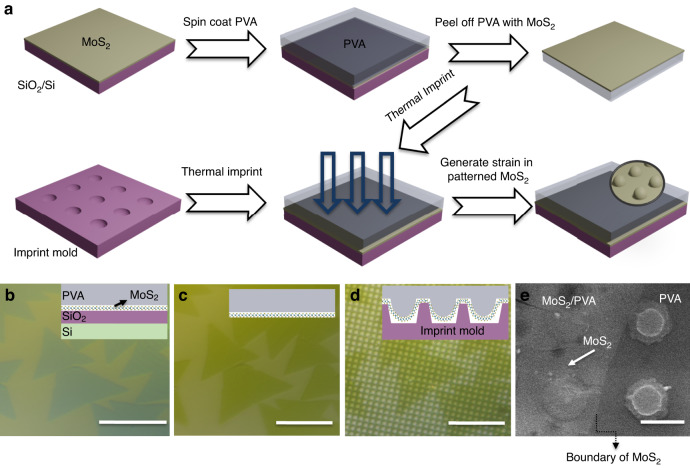
Generation of strain in 2D Materials by NISE. **a** Schematic illustration of the NISE method. Optical images of MoS_2_ monolayers (**b**) before transfer, (**c**) after PVA transfer, and (**d**) imprint with insets showing the cross-sectional schematics. (**e**) SEM image of PVA with and without MoS_2_ after NISE. The scale bars in (**b**–**d**) are 10 µm and the scale bar in (**e**) is 500 nm

We begin the strain engineering process by transferring CVD-grown MoS_2_ sheets onto a PVA substrate. PVA is a thermoplastic polymer with a glass transition temperature (Tg) of 80 °C for nanoimprint lithography, and nanoimprint lithography can enable efficient strain transfer to 2D materials due to the strong interaction between PVA and 2D materials^37–39^. The entire transfer process of MoS_2_ is schematically depicted in Fig. 1a. The PVA was spin-coated onto the target MoS_2_. Afterward, a heating process was performed at 70 °C for 5 min to solidify the PVA layer and further increase the adhesion between MoS_2_ and PVA. After cooling to room temperature, the PVA/MoS_2_ stack was peeled off using tweezers. The strong intercoupling between MoS_2_ and PVA against the weak interaction between MoS_2_ and SiO_2_ ensure the reliable transfer of high-quality MoS_2_^37^. Then, the as-transferred MoS_2_ was placed onto an OrmoStamp mold with the designed structures for nanoimprinting. At a temperature above the Tg of PVA (i.e., 100 °C), PVA becomes soft and then reshapes according to the pattern of the mold with the application of pressure, causing the layer of MoS_2_ to deform. The NISE process is accomplished after a complete cooldown. This NISE technique enables the generation of controllable strain in 2D materials embedded within sandwich structures; this promotes the seamless incorporation of strain into devices based on 2D materials. In contrast to the previously reported nanoimprint strain engineering methods that require the removal of the mold from 2D materials after cooling, our method eliminates the need for such a separation process. This pioneering approach could remarkably minimize the polymer’s elastic response and reduce the risk of potential damage mentioned in previous studies, thereby largely preserving the strain generated in 2D materials. As shown in Fig. 1b, c, MoS_2_ triangular grains grown on a SiO_2_/Si substrate are perfectly transferred onto PVA with highly preserved initial morphologies of the MoS_2_. Figure 1d shows the detailed image of MoS_2_ after NISE, which clearly shows the structures generated in MoS_2_ by the NISE method. This indicates the presence of deformation and strain in the MoS_2_ layer after NISE. The scanning electron microscopy (SEM) image in Fig. 1e depicts the PVA with and without MoS_2_ covered after NISE.

Figure 1d, e shows that the nanopillars were successfully replicated on PVA, exhibiting a period of 1.25 µm and a depth of 90 nm. However, in the presence of the MoS_2_ layer, the spacing between the nanopillars covered with MoS_2_ tends to be greater than that between the nanopillars in the pure PVA region, as shown in Figure S1, since the MoS_2_ sheet acts as a resisting layer, preventing the deformation of PVA. The difference between the bare PVA and MoS_2_/PVA regions is further verified by the corresponding topography profiles in Figure S1, which provide evidence for the existence of MoS_2_.

### Spatial variation of the strain profile of MoS_2_ verified by Raman and PL spectroscopy

**Fig. 2:**
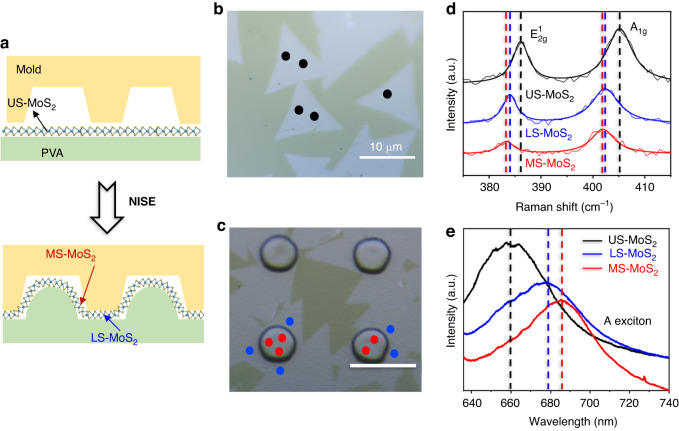
NISE of the monolayer MoS_2_. **a** Schematic illustration of the deformation of MoS_2_ before and after the NISE process. Optical microscope images of the MoS_2_ before NISE (**b**) and after NISE (**c**). The black, blue, and red dots on (**b**) and (**c**) mark the locations where Raman and PL measurements are taken to assess the strain magnitudes. **d** Representative Raman spectra and (**e**) representative PL spectra of MS-MoS_2_, LS-MoS_2_, and US-MoS_2_. The scale bars are 10 μm in (**b**, **c**)

To further clarify the process of strain generation during NISE, Fig. 2a shows schematic diagrams demonstrating the deformation of MoS_2_ before and after the NISE process. An OrmoStamp mold with hole arrays, having a period of 15 µm, a hole diameter of 6 µm, and a depth of 900 nm, was employed for nanoimprint lithography. Before NISE, MoS_2_ was on PVA without a structured morphology, as shown in Fig. 2b. After the implementation of the NISE method, the softened PVA fills the OrmoStamp mold and duplicates the structures of the mold, resulting in the deformation of MoS_2_. Figure 2c shows an optical microscopic view of MoS_2_/PVA after NISE, where replicated pillar arrays can be distinctly observed. As illustrated in Fig. 2a, the MoS_2_ on the pillars experiences large strain due to the pronounced deformation, and the strain gradually decreases toward the region situated between the pillars. The spatially varying strain distribution is further verified by Raman and PL spectroscopy. Figure 2d shows the typical Raman spectra of the most strained-MoS_2_ (MS-MoS_2_) on the pillar, less strained-MoS_2_ (LS-MoS_2_) between the pillars, and unstrained-MoS_2_ (US-MoS_2_) before the NISE method. US-MoS_2_ has the typical Raman spectrum of monolayer MoS_2,_ with two prominent Raman peaks ${\text{E}}_{2\text{g}\,}^{1}$ and A_1g_ centered at 386 cm^−1^ and 405 cm^−1^, respectively; this result confirms the single-layer thickness of MoS_2_^25,40^. The MoS_2_ on the pillars after NISE redshifts for both the ${\text{E}}_{2\text{g}\,}^{1}$ and A_1g_ modes, with average shift magnitudes of 3 and 3.45 cm^−1^, respectively, calculated from the Raman measurements of five different red points in Fig. 2c. However, the MoS_2_ located between the pillars exhibits fewer redshifts for both peaks, with average shift magnitudes of 2 and 2.7 cm^−1^ calculated from the five different blue points. The positions of Raman peaks are sensitive to strain, and the peaks of MoS_2_ under tensile strain exhibit a redshift due to strain-induced phonon softening, which could be used to quantify the strain magnitude of MoS_2_^41,42^. From an overall fit to the redshifts of the ${\text{E}}_{2\text{g}\,}^{1}$ peak^43,44^, 0.58% and 0.4% of the biaxial tensile strains are estimated to be detected on average by the 1 µm diameter laser beam in MS-MoS_2_ and LS-MoS_2_, respectively.

In this study, larger redshifts in the out-of-plane mode A_1g_ peak are observed after the NISE process. These results indicate that the induced strain is predominantly out-of-plane, which accounts for the greater redshifts in the A_1g_ mode and is in agreement with similar observations from other studies^22^. Although doping can also affect the Raman peak, our experimental results support the predominant role of strain in the Raman peak shift. Control experiments using a flat imprint mold yielded no substantial Raman shifts, as demonstrated in Figure S9, indicating that surface contact does not necessarily result in doping, and the observed shifts are more likely caused by the applied strain during the imprinting process. Furthermore, the application of varied imprint pressures results in different strain levels, directly correlating with the observed shifts in the Raman spectra, which will be discussed in the subsequent section. The observed redshift in the less doping-sensitive mode ${\text{E}}_{2\text{g}\,}^{1}$^45,46^ provides a reliable metric for assessing strain in monolayer transition metal dichalcogenides (TMDCs), substantiating the effectiveness of the NISE technique in strain modulation.

Figure 2e shows the typical PL spectra of MS-MoS_2_, LS-MoS_2_ after NISE, and US-MoS_2_ before NISE. US-MoS_2_ shows a typical PL spectrum of monolayer MoS_2_ with a principal peak at 660 nm (A exciton). After NISE, redshifts are observed in the PL spectra of MS-MoS_2_ and LS-MoS_2_, with average shift magnitudes of 25.5 and 16 nm, respectively. The redshifts in the PL spectra obtained under tensile strain are attributed to the decrease in the band gap induced by strain, which agrees with the findings of previous studies^44^. Based on the magnitude of the redshifts^44^, approximately 0.67% and 0.4% of the biaxial tensile strains are measured on average by the 1 µm diameter laser beam in MS-MoS_2_ and LS-MoS_2_, respectively, which aligns with the strain values calculated from the Raman peak shifts.

### Strain modulation of MoS_2_ by the NISE method using different imprint pressures

**Fig. 3:**
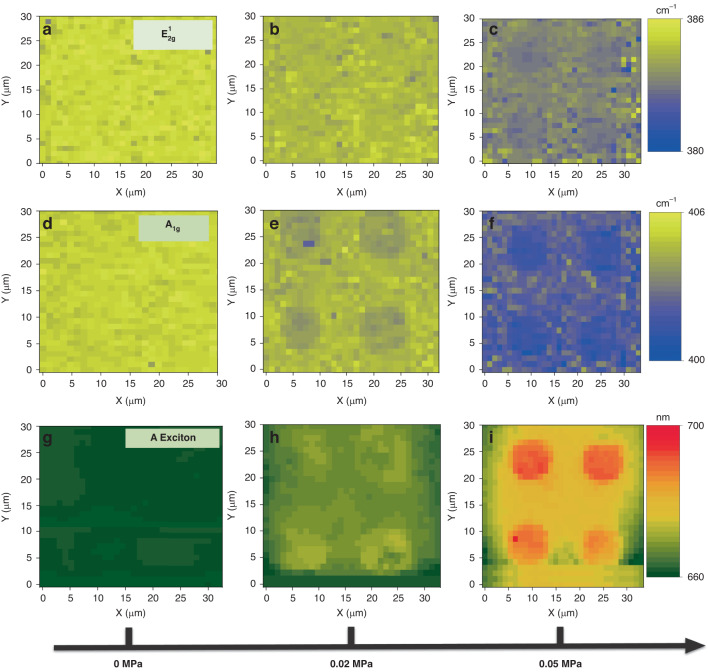
Strain modulation of monolayer MoS2 by NISE method using different pressures. The Raman ${\text{E}}_{2\text{g}}^{1}$ peak wavenumber mapping of MoS_2_ under the pressure of (**a**) 0 MPa, (**b**) 0.02 MPa, and (**c**) 0.05 MPa. The Raman A_1g_ peak wavenumber mapping of MoS_2_ under the pressure of (**d**) 0 MPa, (**e**) 0.02 MPa, and (**f**) 0.05 MPa. The PL A exciton peak wavelength mapping of MoS_2_ under the pressure of (**g**) 0 MPa, (**h**) 0.02 MPa, and (**i**) 0.05 MPa

In nanoimprint lithography, imprint pressure serves as a key determinant of the extent of imprint deformation. By controlling the imprint pressure, a partial imprint of PVA can be achieved, resulting in MoS_2_ with different levels of strain. Herein, we further investigated the effect of imprint pressure on the strain magnitude of MoS_2_ with the NISE method. We adopted the same OrmoStamp mold with hole arrays and a full-area-coverage monolayer of MoS_2_. The spatially varying strain profiles of MoS_2_ are confirmed by Raman and PL mapping. The corresponding positions of the Raman ${\text{E}}_{2\text{g}\,}^{1}$ and A_1g_ and the PL peaks collected from MoS_2_ under different imprint pressures were examined to illustrate the strain modulation capability of the NISE method. The Raman mappings of the ${\text{E}}_{2\text{g}\,}^{1}$ and A_1g_ peak positions of MoS_2_ under a pressure of 0.05 MPa in Fig. 3c, f show that the strain of MoS_2_ varies periodically according to the imprint mold. The blue (lower wavenumber) and yellow color (higher wavenumber) indicate that the tensile strain reaches a maximum on the pillars and then gradually decreases to a minimum in the region between the pillars. Notably, a periodically varying strain pattern is consistently observed across the entire scanned area (900 µm^2^). To provide a clearer understanding of the periodic strain profile in MoS_2_, the variations in the ${\text{E}}_{2\text{g}\,}^{1}$ and A_1g_ peak positions over two periods are depicted in Fig. S3d, e. The maximum strain in MoS_2_ in the pillar region results from significant deformation of MoS_2_/PVA as they fill the gap between MoS_2_ and the mold, while the decreased strain of MoS_2_ in the interpillar region could be attributed to sliding between PVA and MoS_2_. We also analyzed the strain profile of MoS_2_ by scanning PL spectroscopy. Similar to the Raman mapping results, the mapping of the PL peak position shows a periodic distribution following the patterns of the imprint mold (Fig. 3i). The variations in the PL peak positions over two periods are also extracted and plotted in Fig. S3f to demonstrate the periodic variations consistent with the mold. All these findings conclusively demonstrate the ability of the NISE method to introduce a spatially varying strain profile into a MoS_2_ sheet that aligns with the imprint mold pattern.

To further investigate the effect of imprint pressure on the strain magnitude of the MoS_2_, Raman maps of the ${\text{E}}_{2\text{g}}^{1}$ and A_1g_ peak positions of the MoS_2_ sheets with different imprint pressures (i.e., 0 and 0.02 MPa) are shown in Fig. 3a, b and Fig. 3d, e. Before NISE (i.e., 0 MPa), the peak positions are clearly uniformly distributed over the 30×30 µm scanning area in Fig. 3a, d, while the mappings show a periodic distribution according to the imprint mold after the application of the NISE method. Similarly, the mappings exhibit more redshifted peak positions on the pillars and slight redshifts between the pillars. Moreover, the shift magnitude increases with increasing imprint pressure. Compared with those in Fig. 3b, e, the strain profiles generated by the NISE method are more evident in Fig. 3c, f, with more significant redshifts occurring over the scanning area, especially those on the pillars. This occurs because a higher imprint pressure can cause more severe deformation, leading to greater tensile strain being generated in the MoS_2_ sheet. The evolutions of the Raman peak positions of MoS_2_ over two periods under different pressure levels are shown in Fig. S4a, b; here, the characteristic redshift behavior of the Raman peak with increasing pressure is clearly observed. The strain modulation capability of the NISE method is further supported by PL characterization of the same area. Figure 3g–i depicts the variations in the PL A peak position mapping with increasing pressure. In contrast to the uniform mapping of the PL peak position at 0 MPa, the PL peak position mapping shows a periodic distribution and more significant redshift behavior under higher pressure, reflecting the changes in the band gap under different imprint pressures. The evolutions of the PL peak positions of MoS_2_ at different pressure levels are shown in Fig. S4c. The optical images in Fig. S2 show the diverse topographies of MoS_2_ under different pressure levels, clearly revealing various degrees of deformation. Overall, the results of scanning Raman and PL spectroscopy confirm the generation of spatially varying strain profiles of MoS_2_ that follow the structures of the imprint mold, and the strain magnitude could be further manipulated by different imprint pressures.

### Finite element simulations of 3D straining of MoS_2_

**Fig. 4:**
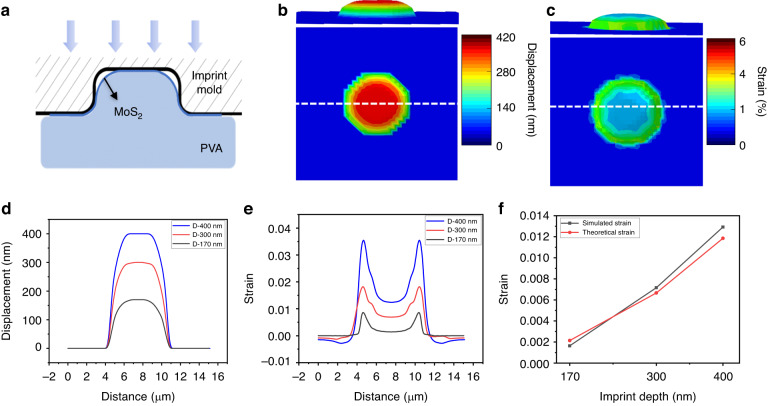
Simulated mechanical response of MoS2 after NISE. **a** Simulated structure of the imprint mold/MoS_2_/PVA during the NISE process. The 3D display of (**b**) displacement distribution and (**c**) strain distribution of the MoS_2_ layer after NISE with the top panel showing the cross-sectional view. The extracted (**d**) displacement and (**e**) strain profile of MoS_2_ after NISE with imprint depth of 170 nm, 300 nm, and 400 nm along the white dashed line. **f** Comparison of the strain values calculated from theoretical and simulated results at various imprint depths

To analyze the straining behavior of MoS_2_ during the NISE process, finite element simulations were carried out in Abaqus. The mechanical responses of the strained MoS_2_ imprinted by a cavity mold were studied. 3D finite element models were employed to analyze the deformation mechanism of the 2D materials during the NISE process. To simplify the simulations, several assumptions are made as follows: (a) the imprint is considered to be a rigid body with no deformation; (b) the imprint resist (i.e., PVA) is an incompressible and hyperelastic polymer; (c) MoS_2_ is modeled as a single-layer membrane without bending stiffness; and (d) no separation exists between MoS_2_ and PVA or between MoS_2_ and the mold once they come into contact with each other during the NISE process. Since the Tg of PVA is 85 °C, all simulations are performed at 120 °C, which is consistent with the experimental conditions. Figure 4a shows a schematic cross-sectional view of the simulated imprint mold/MoS_2_/PVA assembly. In this configuration, the cavity of the mold is occupied by PVA, which leads to the deformation of the MoS_2_ membrane coordinated with the flow of the imprint resist. A circular mold with a 6000 nm hole diameter and a 400 nm depth is initially utilized to simulate the mechanical response of MoS_2_ during NISE. As the temperature surpasses the Tg, deformation and bulging of the 2D sheet are induced by the pressurized fluidic imprint resist. The simulation process is complete when the cavity is entirely filled with imprint resistance. Figure 4b, c depicts the 3D visualization of the displacement and strain profile of the MoS_2_ layer after NISE. Figure 4d plots the distribution of the displacement of MoS_2_ with an imprint depth of 400 nm in blue after NISE. The maximum displacement of MoS_2_ at the center of the cavity reaches nearly 400 nm, replicating the dimensions of the imprint mold. The extracted displacement profile of MoS_2_ shows that the peak at the center reaches the cavity ceiling before lateral filling occurs, and then, the MoS_2_ along with the polymer spreads laterally to conform to the mold; this results in locally high strain around the sidewall of the cavity, as shown in Fig. 4e. This deformation mode is similar to that of an imprinted polymer during the thermal nanoimprinting process reported in previous studies^47–49^. The straining capability of NISE is further investigated by simulating the imprinted MoS_2_ with various imprint depths. Figure 4d shows the evolution of the displacement of MoS_2_ with the peak traveling to different heights (i.e., 170, 300, and 400 nm). The evolution of the von Mises strain is shown in Fig. 4e. The strain clearly increases with increasing imprint depth. Similarly, a region of locally high strain appears near the sidewall of the cavity, and this effect becomes more pronounced as the imprint height increases. Other imprint parameters, such as the imprint temperature, may also contribute to the strain generated by the NISE method since the viscosity of the imprint resist is largely dependent on the imprint temperature. 2D materials undergo a more severe degree of deformation under conditions that are more favorable to polymer flow. Consequently, the magnitude of strain introduced to MoS_2_ increases with increasing imprint temperature.

To further understand the straining behavior of MoS_2_, the biaxial strain at the center of the membrane could be calculated following the Hencky model^50^ for large deflections of a clamped, circular isotropic membrane under uniform pressure. The strain at the center is derived as $\varepsilon =\sigma \left({\rm{\mu }}\right){\left(\frac{\delta }{\text{a}}\right)}^{2}$, where σ(μ) is a constant that depends on Poisson’s ratio μ, *δ* is the deflection height at the center and $\text{a}$ is the radius of the membrane. The value of σ (μ) is used as 0.709^43^ with μ = 0.29^51^ for MoS_2_. Therefore, we can estimate the strain at each deflection height by using the Hencky model. In the case of MoS_2_ with a deflection height of 400 nm and a radius of 3000 nm, the strain reaches 1.3%, which is consistent with the strain at the center of the cavity in the simulations. These findings strongly align our theoretical calculations and simulation results across various imprint depths, as demonstrated in Fig. 4f, which highlights the robustness of our simulation model.

### NISE extended to other 2D materials with different strain distributions

**Fig. 5:**
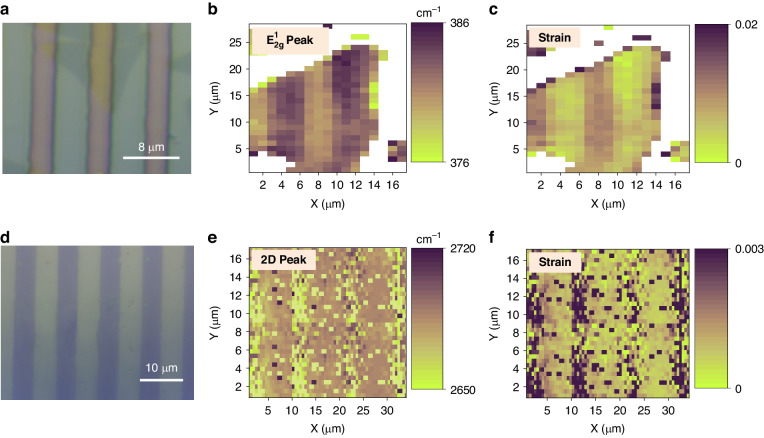
NISE extended to other 2D materials with different strain distributions. **a** Optical image of the strained triangular monolayer MoS_2_ after the NISE method with the grating profile. **b** Corresponding Raman mapping showing the ${\text{E}}_{2\text{g}}^{1}$ peak wavenumber and the (**c**) strain mapping estimated from the ${\text{E}}_{2\text{g}}^{1}$ peak shift. **d** Optical image of the strained monolayer graphene after NISE with the grating profile. **e** Corresponding Raman mapping of the 2D peak position and the (**f**) strain mapping estimated from the 2D peak shift

The deformation of 2D materials follows the shape of the mold, enabling easy control of the strain distribution. This facile and reliable strain engineering technique can also be applied to other 2D materials as long as they can be transferred onto a thermal plastic substrate for thermal imprinting. To demonstrate this, we employ a patterned SiO_2_/Si mold with microtrenches (a period of 8 µm, trench width of 3 µm, and depth of 200 nm) to imprint monolayer MoS_2_ triangles. The strain distribution was also examined using Raman and PL spectroscopy. Figure 5a shows the strained triangular monolayer MoS_2_ after NISE. Corresponding to the patterns of the mold, the mapping of the ${\text{E}}_{2\text{g}\,}^{1}$ peak wavenumber in Fig. 5b shows a profile that follows the mold’s structures in a systematic manner. MoS_2_ located in the trough region is represented by a yellow color (lower wavenumber) with a notable redshift of 5 cm^−1^. On the other hand, MoS_2_ in the peak region shows a purple color (higher wavenumber), with a redshift of 2.6 cm^−1^. The corresponding strain mapping evaluated from the redshift of the ${\text{E}}_{2\text{g}\,}^{1}$ peak shows the periodic strain distribution with respect to the imprint mold (Fig. 5c). Calculations based on the redshift indicate that the strain experienced by MoS_2_ in the trough region increases to 0.97%. The grating strain profile of MoS_2_ is further supported by scanning PL spectroscopy, as shown in Fig. S7. An average redshift of 37 nm and 23 nm in the PL peak is obtained in the trench region and top region, respectively. A tensile strain of 0.97% is calculated in the trough region according to the redshift of the PL peak, which coincides with the strain estimated from the Raman peak shift.

In addition to MoS_2_, the NISE method can be extended to other 2D materials. To demonstrate this, we applied this approach to introduce strain to monolayer graphene. The main features in the Raman spectra of the monolayer CVD-grown graphene are the G and 2D peaks, located at 1583 and 2696 cm^−1^, respectively, before NISE (Fig. S5). With graphene transferred onto PMMA, a grating strain profile could be generated on graphene via the NISE method. The imprint mold utilized has a grating pattern with a period of 10 µm, a trench width of 5 µm, and a depth of 250 nm. Figure 5d shows an optical microscopy image of the PMMA/graphene/structured SiO_2_ stack after NISE. The distributions of the phonon wavenumbers in the 2D band in Fig. 5e and in the G band in Fig. S8 vary consistently with the pattern of the mold, showing a redshift of approximately 37 and 10 cm^−1^, respectively, in the most strained region. The strain mapping in Fig. 5f further depicts the strain mapping of the graphene evaluated from the 2D peak shift. Fig. S6 shows the Raman spectra of the most strained graphene (MS-graphene) in the trough region, less strained graphene (LS-graphene) in the peak region, and unstrained graphene (US-graphene). The figure clearly shows the variations in the redshift and peak splitting behavior of the Raman peaks in different regions subjected to varying strain levels, which effectively elucidates how the strain profiles of the 2D materials spatially vary as guided by the imprint mold. Therefore, our approach has demonstrated its advantages as an efficient and versatile strategy for generating a wide range of strain profiles in 2D materials. Furthermore, our NISE process can be adapted to utilize alternative polymers. For instance, inorganic polymers such as hydrogen silsesquioxane (HSQ) can be used as substitutes for PVA. HSQ has a decreased density of dangling bonds and may serve as a more suitable interface for 2D materials in certain applications, thus mitigating the potential risks associated with PVA. Being scalable and cost-effective, our strategy has significant potential for applications in devices integrating strain with 2D materials.

## Conclusion

In summary, we have reported an effective strain engineering method for generating controllable and spatially modulated strains on 2D materials by using thermal imprint lithography. The ability of the NISE method to generate spatially varying strain distributions was evaluated by scanning Raman and PL spectroscopy. Continuous tuning of the strain magnitude and generation of various strain distributions of 2D materials were successfully achieved via the NISE strategy. Compared with other strain engineering techniques, our method can introduce more deterministic strain at low cost and high throughput. Additionally, our approach minimizes damage to 2D materials and can potentially maximize the strain in 2D materials through the careful design of the imprint mold and imprint conditions. The 2D layer is sandwiched between the imprint resist and mold and can sustain the strain because the strain conforms tightly to the designated structures. Our process does not require any special equipment or conditions to preserve the strain in the 2D materials; thus, our process is a more viable option for strain-engineered 2D material devices. Moreover, our method can also be extended for the introduction of strain into other 2D materials transferred on thermal plastic substrates, which has promise for the fabrication of strain-integrated 2D material devices with improved performance. Our newly developed strain engineering approach enables the generation of spatially modulated strains in a more controllable and sustainable way. Since the NISE method is a promising method for precisely modulating the properties of 2D materials, this approach will play an instrumental role in harnessing their distinctive properties for the advancement of next-generation 2D material-based devices.

## Experimental section

### Transfer and straining of MoS_2_

CVD-grown MoS_2_ on a SiO_2_ substrate (purchased from MetaTest Corporation) was spin-coated with a layer of PVA at 500 RPM for 30 s. After baking the sample at 70 °C for 5 min, the MoS_2_/PVA was peeled off using tweezers. Subsequently, the PVA-transferred MoS_2_ was placed on the imprint mold for NISE, ensuring that the MoS_2_ side was in good contact with the imprint mold. With temperature at 120 °C (above the Tg of PVA (80 °C)), MoS_2_/PVA was deformed and imprinted under pressure for 15 min. Last, the whole stack was cooled to room temperature while maintaining the pressure supply.

### Transfer and straining of graphene

Single-layer graphene grown on Cu foil (purchased from 2D semiconductors) was spin-coated with a layer of PMMA at 3000 RPM for 60 s. Then, the sample was heated on a hot plate at 80 °C for 5 min to remove the solvent. Afterward, the copper foil was etched away by immersion in a FeCl_3_ solution. After the PMMA/graphene was rinsed in deionized water for 30 min, the target substrate was removed. The following procedures for the straining of graphene are the same as those for MoS_2_ according to the NISE method.

### Imprint mold fabrication

The OrmoStamp imprint molds were fabricated by a conventional photolithography process followed by the transfer of the photoresist pattern. First, a Si substrate was spin-coated with a layer of AZ1505 photoresist and then baked at 100 °C for 1 min. When the Si substrates were cooled to room temperature, photolithography was conducted by exposing the photoresist to 365 nm UV light via a photolithography machine (URE 2000/35, Chinese Academy of Sciences, China) for 11 s. The photoresist was then developed by immersing and stirring the mixture in developer solution (1:4 diluted AZ351B) for 1 min. Then, the patterns of the photoresist were transferred into OrmoStamp by adding the OrmoStamp liquid dropwise (Micro Resist Technology GmbH, Germany) on the photoresist sample. A glass substrate with a layer of spin-coated OrmoPrime was then gently placed onto the OrmoStamp liquid. After the photoresist mold was completely filled with OrmoStamp, the OrmoStamp was cured by 405 nm UV light at a dose of 1000 mJ/cm^2^. After the OrmoStamp was separated from the photoresist and the OrmoStamp was thoroughly cleaned with acetone and isopropanol, the OrmoStamp mold for the NISE method was obtained. The SiO_2_/Si molds with microtrenches were fabricated by photolithography followed by ICP etching.

### Materials characterization

PL and Raman spectra were collected by using a spectral scanning test system (MetaTest ScanPro Advance) with an excitation laser line of 532 nm at room temperature in an ambient air environment. The laser power was kept below 1 mW to avoid damage to MoS_2_ due to thermal heating, and the size of the laser spot was ≈1 µm. Gratings with 1800 lines/mm and 500 lines/mm were used for the Raman and PL measurements, respectively. The morphologies of the 2D materials were observed by optical microscopy, SEM (Hitachi S-4800), and AFM (Bruker Multimode 8-HR).

### Finite element simulations of the straining behavior of MoS_2_ during NISE

A three-dimensional (3D) finite element model with an imprint mold/MoS_2_/PVA structure is employed in Abaqus to predict the deformation of MoS_2_ during the NISE process. The imprint mold is considered to be a rigid body that cannot be deformed under an external force. MoS_2_ is assumed to be a single-layer membrane, and PVA behaves as an incompressible and nonlinear hyperelastic polymer. The membrane elements provide strength in the plane of the element but have no bending stiffness; these are appropriate for representing the mechanical behavior of a 2D material under strain. Then, we define the skin reinforcements as MoS_2_ such that it is similar to a thin film draped over the PVA surface. There is no sliding and separation between MoS_2_ and PVA or between MoS_2_ and the imprint mold once they come in contact with each other. A constant force is applied to the imprint mold with confined vertical displacement on the bottom surface of the PVA, and a symmetric boundary condition is applied. The NISE process is investigated above the Tg of PVA.

### Supplementary information

Supplemental materials

## References

[1] Manzeli, S., Ovchinnikov, D., Pasquier, D., Yazyev, O.V. & Kis, A. 2D transition metal dichalcogenides. *Nat. Rev. Mater.* **2** (2017).

[2] Wang P, Duan X (2021). Probing and pushing the limit of emerging electronic materials via van der Waals integration. MRS Bull..

[3] Wu, Y., Li, D., Wu, C. L., Hwang, H. Y. & Cui, Y. Electrostatic gating and intercalation in 2D materials. *Nat. Rev. Mater.* **0123456789** (2022).

[4] Bafekry A (2021). Van der Waals heterostructure of graphene and germanane: Tuning the ohmic contact by electrostatic gating and mechanical strain. Phys. Chem. Chem. Phys..

[5] Qi D (2019). Continuously tuning electronic properties of few-layer molybdenum ditelluride with in situ aluminum modification toward ultrahigh gain complementary inverters. ACS Nano.

[6] Sun J (2020). Lateral 2D WSe_2_ p–n homojunction formed by efficient charge-carrier-type modulation for high-performance optoelectronics. Adv. Mater..

[7] Kumar V, Dey A, Thomas S, Asle Zaeem M, Roy DR (2021). Hydrogen-induced tunable electronic and optical properties of a two-dimensional penta-Pt_2_N_4_ monolayer. Phys. Chem. Chem. Phys..

[8] Pu C, Yu J, Yu R, Tang X, Zhou D (2019). Hydrogenated PtP_2_ monolayer: Theoretical predictions on the structure and charge carrier mobility. J. Mater. Chem. C. Mater..

[9] Zheng F (2020). Critical stable length in wrinkles of two-dimensional materials. ACS Nano.

[10] Cao K (2020). Elastic straining of free-standing monolayer graphene. Nat. Commun..

[11] Yang S, Chen Y, Jiang C (2021). Strain engineering of two‐dimensional materials: Methods, properties, and applications. InfoMat.

[12] Du J (2021). Strain engineering in 2D material-based flexible optoelectronics. Small Methods.

[13] Peng, Z., Chen, X., Fan, Y., Srolovitz, D. J. & Lei, D. Strain engineering of 2D semiconductors and graphene: from strain fields to band-structure tuning and photonic applications. *Light Sci. Appl.* **9**10.1038/s41377-020-00421-5 (2020). 10.1038/s41377-020-00421-5 PMC7680797 33298826

[14] Hosseini M, Elahi M, Pourfath M, Esseni D (2015). Strain-Induced modulation of electron mobility in single-layer transition metal dichalcogenides MX_2_ (M= Mo, W; X=S, Se). IEEE Trans. Electron Devices.

[15] Duerloo KAN, Li Y, Reed EJ (2014). Structural phase transitions in two-dimensional Mo-and W-dichalcogenide monolayers. Nat. Commun..

[16] Desai SB (2014). Strain-induced indirect to direct bandgap transition in multilayer WSe_2_. Nano Lett..

[17] Maiti R (2020). Strain-engineered high-responsivity MoTe_2_ photodetector for silicon photonic integrated circuits. Nat. Photonics.

[18] Datye IM (2022). Strain-Enhanced Mobility of Monolayer MoS_2_. Nano Lett..

[19] Ng HK (2022). Improving carrier mobility in two-dimensional semiconductors with rippled materials. Nat. Electron.

[20] Blundo, E., Cappelluti, E., Felici, M., Pettinari, G. & Polimeni, A. Strain-tuning of the electronic, optical, and vibrational properties of two-dimensional crystals. *Appl. Phys. Rev.* **8**10.1063/5.0037852 (2021).

[21] Dai Z, Liu L, Zhang Z (2019). Strain engineering of 2D materials: issues and opportunities at the interface. Adv. Mater..

[22] Hsieh Y-C (2023). Engineering the strain and interlayer excitons of 2D materials via lithographically engraved hexagonal boron nitride. Nano Lett..

[23] Regan EC (2022). Emerging exciton physics in transition metal dichalcogenide heterobilayers. Nat. Rev. Mater..

[24] Jiang Y, Chen S, Zheng W, Zheng B, Pan A (2021). Interlayer exciton formation, relaxation, and transport in TMD van der Waals heterostructures. Light Sci. Appl.

[25] Li F, Shen T, Xu L, Hu C, Qi J (2019). Strain improving the performance of a flexible monolayer MoS_2_ photodetector. Adv. Electron Mater..

[26] John AP, Thenapparambil A, Thalakulam M (2020). Strain-engineering the Schottky barrier and electrical transport on MoS_2_. Nanotechnology.

[27] Liu Y, Huang Y, Duan X (2019). Van der Waals integration before and beyond two-dimensional materials. Nature.

[28] Zhang Y, Choi MK, Haugstad G, Tadmor EB, Flannigan DJ (2021). Holey substrate-directed strain patterning in bilayer MoS_2_. ACS Nano.

[29] Hsu CC, Teague ML, Wang JQ, Yeh NC (2020). Nanoscale strain engineering of giant pseudo-magnetic fields, valley polarization, and topological channels in graphene. Sci. Adv..

[30] Nemes-Incze P (2017). Preparing local strain patterns in graphene by atomic force microscope based indentation. Sci. Rep..

[31] Liu X (2020). Thermomechanical nanostraining of two-dimensional materials. Nano Lett..

[32] Zhang H (2016). Kaleidoscopic imaging patterns of complex structures fabricated by laser-induced deformation. Nat. Commun..

[33] Guo CF (2012). Path-guided wrinkling of nanoscale metal films. Adv. Mater..

[34] Du, S. et al. Strain lithography for two-dimensional materials by electron irradiation. *Appl. Phys. Lett.* **120** (2022).

[35] Bensmann, Jannis, et al. Nanoimprint strain-engineering of 2D semiconductors. *arXiv preprint arXiv*:2212.11873 (2022)

[36] Lai YY, Chen PH, Chen CA, Lee YH, Deng H (2022). Single-photon emission from rewritable nanoimprinted localized emitter arrays in atomically thin crystals. ACS Photonics.

[37] Li Z (2020). Efficient strain modulation of 2D materials via polymer encapsulation. Nat. Commun..

[38] Soler-Crespo RA (2019). Atomically thin polymer layer enhances toughness of graphene oxide monolayers. Matter.

[39] Zhao, L. et al. Highly-stable polymer-crosslinked 2D MXene-based flexible biocompatible electronic skins for in vivo biomonitoring. *Nano Energy* **84** (2021).

[40] Hwangbo S, Hu L, Hoang AT, Choi JY, Ahn JH (2022). Wafer-scale monolithic integration of full-colour micro-LED display using MoS_2_ transistor. Nat. Nanotechnol..

[41] Mao J, Wu Z, Guo F, Hao J (2022). Strain-Induced performance enhancement of a monolayer photodetector via patterned substrate engineering. ACS Appl Mater. Interfaces.

[42] Pak S (2020). Strain-engineering of contact energy barriers and photoresponse behaviors in monolayer MoS_2_ flexible devices. Adv. Funct. Mater..

[43] Lloyd D (2016). Band gap engineering with ultralarge biaxial strains in suspended monolayer MoS_2_. Nano Lett..

[44] Li Hong (2015). Optoelectronic crystal of artificial atoms in strain-textured molybdenum disulphide.. Nature commun..

[45] Conley HJ (2013). Bandgap engineering of strained monolayer and bilayer MoS_2_. Nano Lett..

[46] Melnikova-Kominkova Z (2019). Strong and efficient doping of monolayer MoS_2_ by a graphene electrode. Phys. Chem. Chem. Phys..

[47] Sun H, Yin M, Wang H (2017). High aspect ratio nanoimprint mold-cavity filling and stress simulation based on finite-element analysis. Micromachines (Basel).

[48] Rowland HD, King WP (2004). Polymer deformation and filling modes during microembossing. J. Micromech. Microeng..

[49] Jena, R. K., Chen, X., Yue, C. Y. & Lam, Y. C. Rheological (visco-elastic behaviour) analysis of cyclic olefin copolymers with application to hot embossing for microfabrication. *J. Micromech. Microeng.* **21** (2011).

[50] Fichter W (1997). Some solutions for the large deflections of uniformly loaded circular membranes. NASA Tech. Pap..

[51] Cooper, R. C. et al. Nonlinear elastic behavior of two-dimensional molybdenum disulfide. *Phys. Rev. B: Condens Matter Mater. Phys.* **87** (2013).

