# Supplementary material for: Nanoimprint-induced strain engineering of two-dimensional materials
Source: Microsyst Nanoeng. 2024 Apr 8;10:49. doi: 10.1038/s41378-024-00669-6 (PMC11001999; doi:10.1038/s41378-024-00669-6)
Supplement: Supplementary file 1 — Supplemental materials [file 41378_2024_669_MOESM1_ESM.docx]

Supporting Information

Nanoimprint-induced Strain Engineering of Two-dimensional Materials

Chuying Sun, Jiangwen Zhong, Zhuofei Gan, Liyang Chen, Chuwei Liang, Hongtao Feng, Zhao Sun, Zijie Jiang and Wen-Di Li*

^∗^ E-mail addresses: liwd@hku.hk


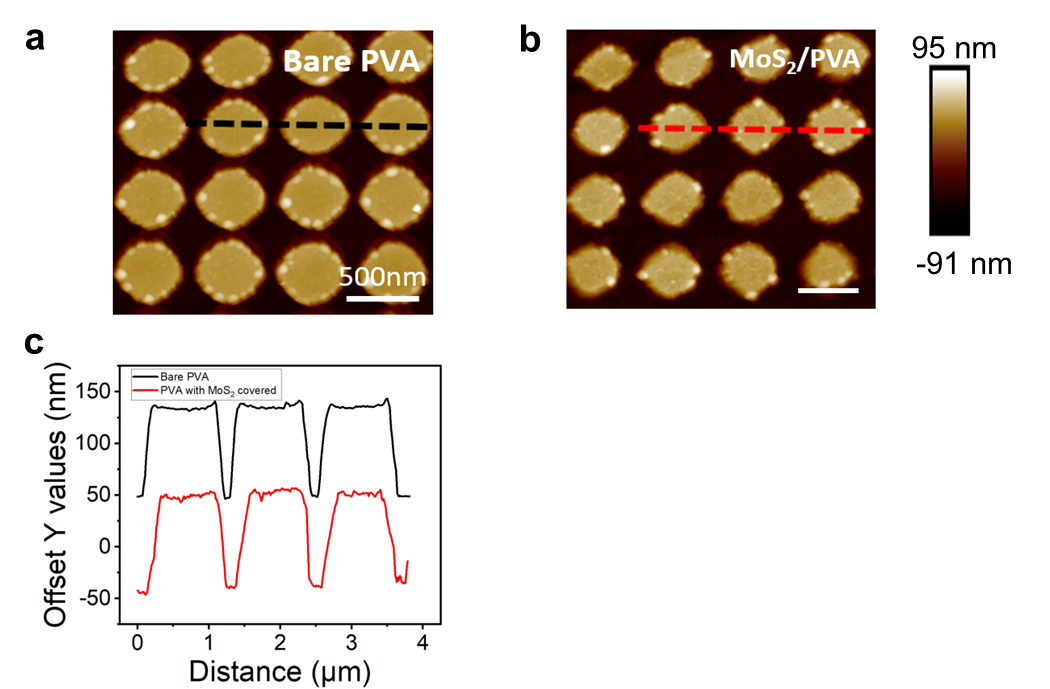


**Figure S1.** Characterization of PVA and MoS_2_/PVA after applying NISE. The topography image of (a) bare PVA and (b) PVA covered with MoS_2_ after NISE. (c) The corresponding topography profiles marked in (a) and (b). The scale bars in (a)(b) are all 500 nm.

**
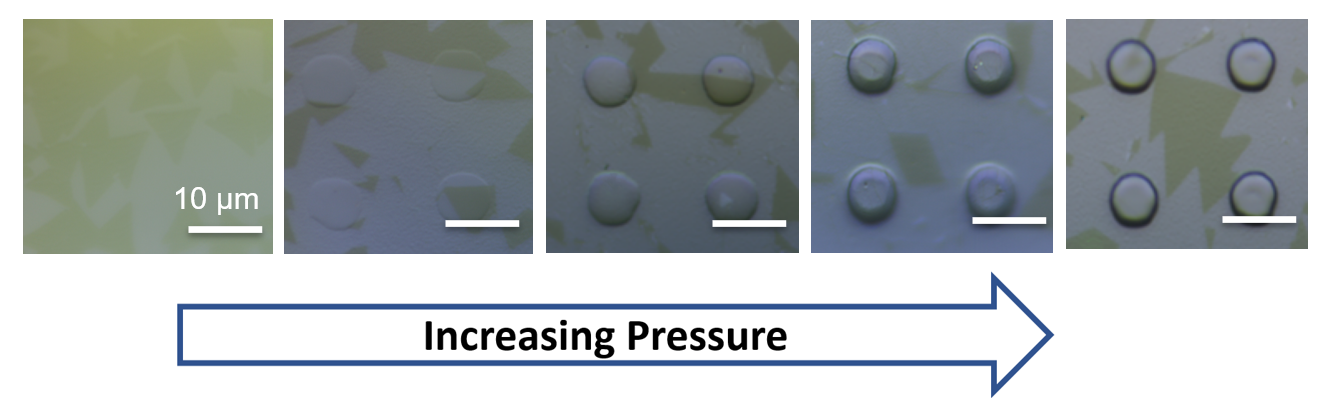
**

**Figure S2.** Optical images of MoS_2_ after NISE under different pressures to show the strain modulation capacity of NISE. The scale bars are all 10 μm.


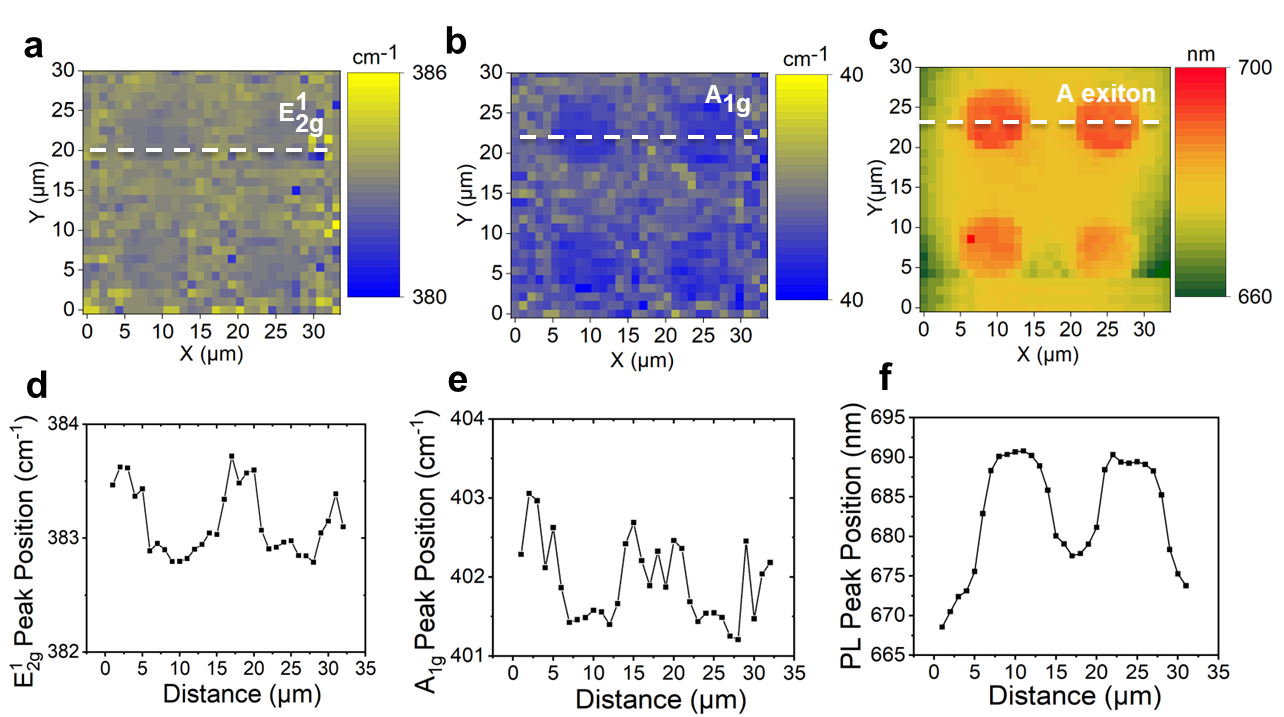


**Figure S3.** Scanning Raman and PL spectroscopy of the strained MoS_2_ by NISE under the pressure of 0.05 MPa. Scanning Raman spectroscopic mapping of the (a) $\text{E}_{\text{2g}}^{\text{1}}$ peak wavemumber and (b) $\text{A}_{\text{1g }}$peak wavenumber. (c) Scanning PL mapping of the A exciton wavelength at the same locations of the sample. Extracted peak position of the (d) $\text{E}_{\text{2g}}^{\text{1}}$ peak, (e) $\text{A}_{\text{1g }}$peak, and (f) A exciton along the white dashed line in (a)(b)(c) ,respectively. The scale bar are all 5 $\text{μm}.$


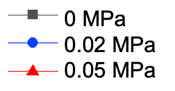

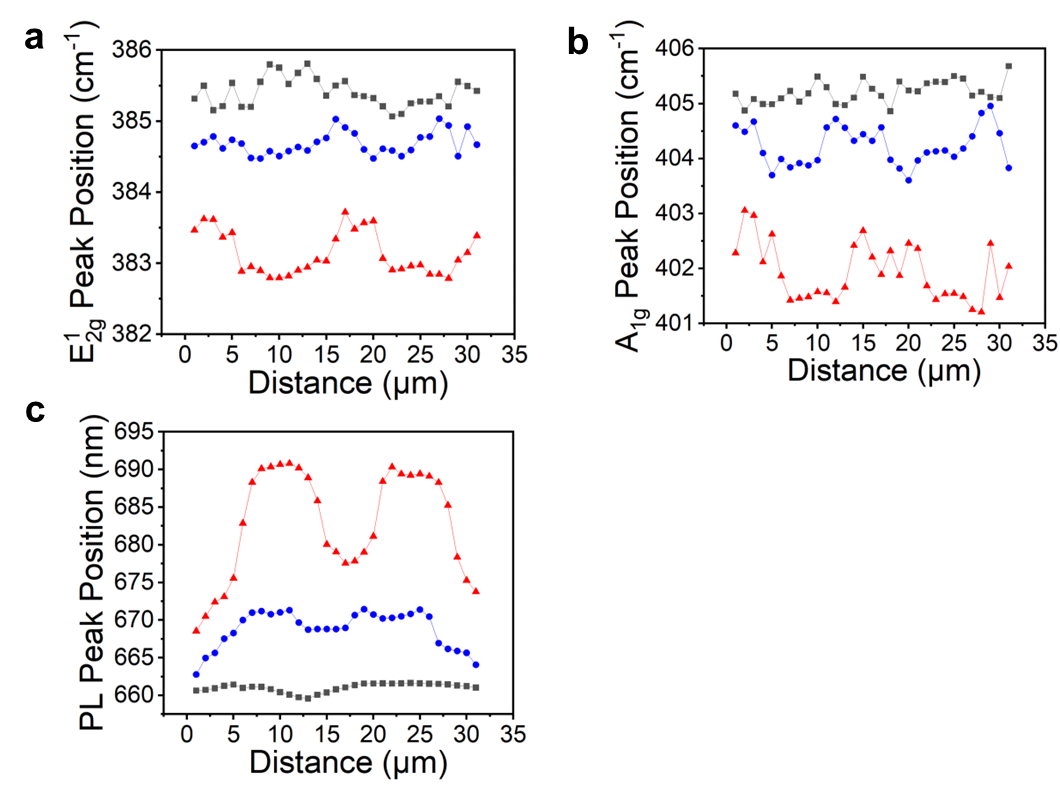


**Figure S4:** The line profile of Raman (a) $\text{E}_{\text{2g}}^{\text{1}}$wavemuber, (b) $\text{A}_{\text{1g }}$ wavenumber and (c) PL A exciton wavelength of MoS_2_ after NISE as a function of distance under various levels of pressure.

**
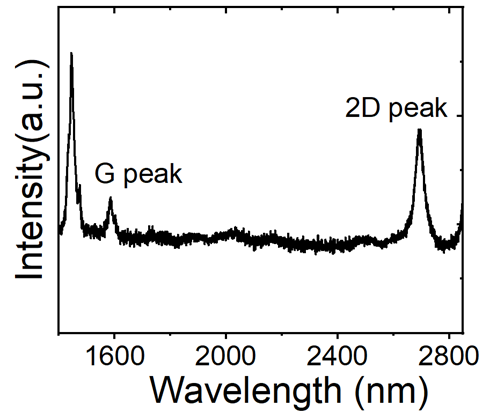
Figure S5:** The Raman spectrum of graphene.


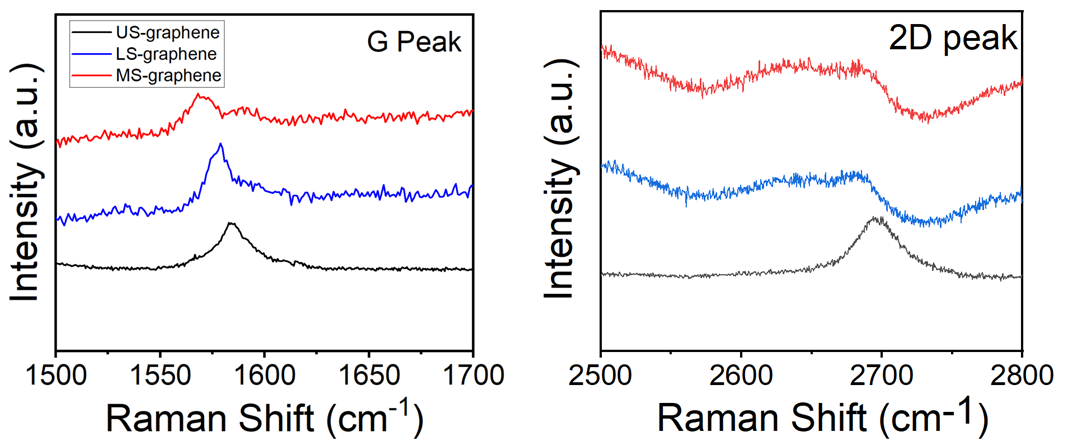


**Figure S6.** The Raman spectrum of unstrained graphene (US-graphene), less-strained graphene (LS-graphene), and most-strained graphene (MS-graphene).


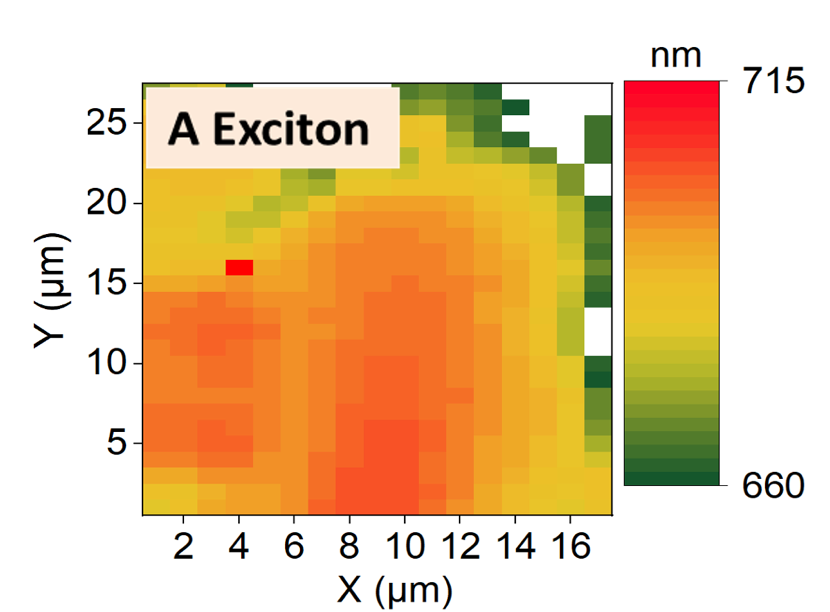


**Figure S7.** The corresponding PL mapping showing the PL peak wavelength of the strained triangular monolayer MoS_2_ in Figure 5a.


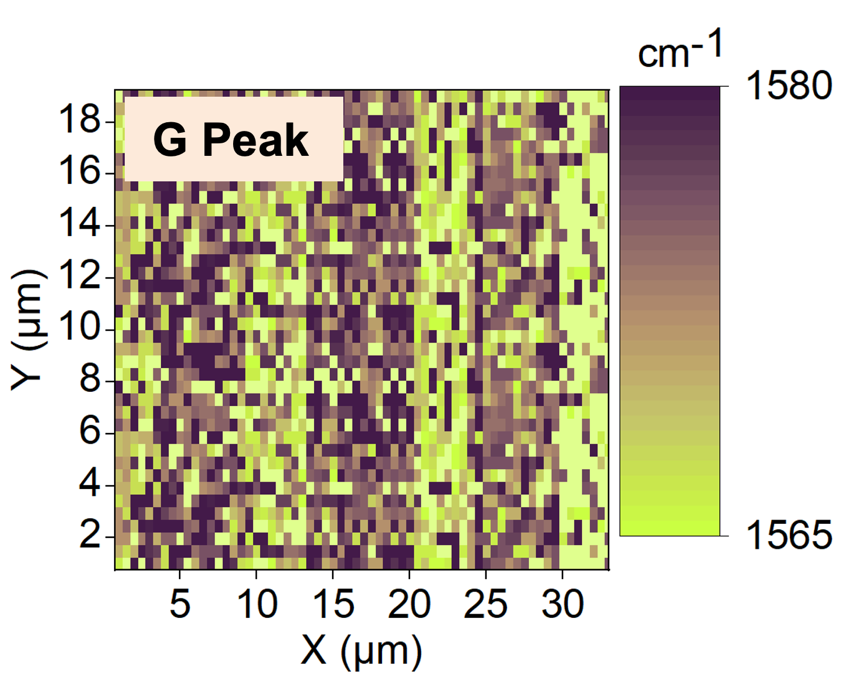


**Figure S8.** The corresponding Raman mapping showing G peak of the strained graphene in Figure 5d .


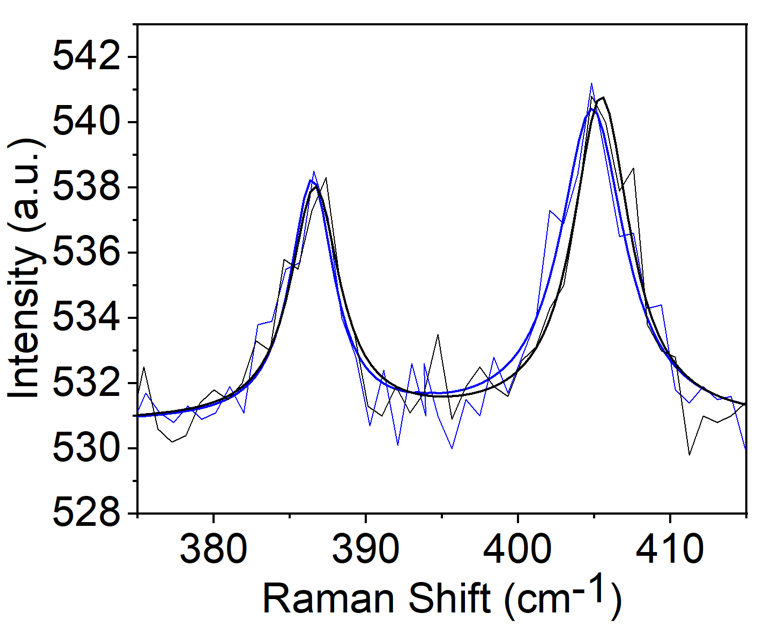


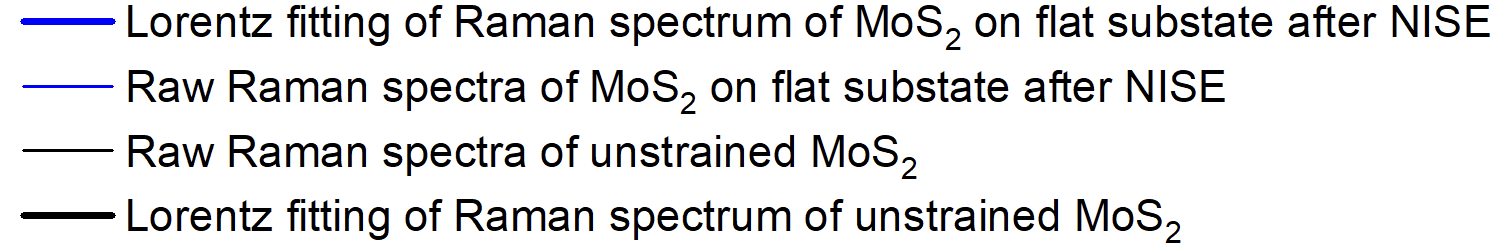


**Figure S9.** The comparison of Raman spectra of MoS_2_ on flat substrate after NISE with the unstrained MoS_2_ which is after transferred on the flat substrate but before NISE. This comparison suggests that the observed Raman shifts are predominantly due to strain rather than doping.
